# Curettage combined with decompression for the treatment of ameloblastoma in children: report of two cases

**DOI:** 10.1186/s12903-024-04126-8

**Published:** 2024-03-22

**Authors:** Chen Xu, Yuhua Hu, Yuhuan Sun, Qiang Shao, Yonghai Song, Jie He

**Affiliations:** 1 Department of Oral and Maxillofacial Surgery, Zibo Central Hospital, Zibo, 255036 China; 2 Department of Oral Pathology, Shanghai Ninth People’s Hospital, Shanghai Jiao Tong University School of Medicine, Shanghai, 200011 China; 3 Department of Oral and Maxillofacial-Head and Neck Oncology, Shanghai Ninth People’s Hospital, Shanghai Jiao Tong University School of Medicine, Shanghai, 200011 China; 4 College of Stomatology, Shanghai Jiao Tong University, Shanghai, China; 5 National Center for Stomatology, Shanghai, China; 6 National Clinical Research Center for Oral Diseases, Shanghai, China; 7 Shanghai Key Laboratory of Stomatology, Shanghai, China; 8 Shanghai Research Institute of Stomatology, Shanghai, China; 9 Shanghai Center of Head and Neck Oncology Clinical and Translational Science, Shanghai, China

**Keywords:** Ameloblastoma, Curettage, Decompression, Benign odontogenic tumor, Children

## Abstract

**Background:**

Ameloblastoma (AM) is the most common benign odontogenic tumor, which is more often detected in the mandible than maxilla, especially the mandibular body and mandibular angle. Pediatric AM is a rare disease, especially in patients aged 10 and younger. Compared with the mainstream osteotomy and reconstructive surgery for adult ameloblastoma, there is more room for discussion in the treatment of pediatric ameloblastoma. The postoperative functional and psychological influence can not be ignored. Especially for children in the period of growth and development, an osteotomy is often challenging to be accepted by their parents. We report two patients with ameloblastoma under 10 years old who are treated with curettage and fenestration, which is a beneficial method for children with ameloblastoma.

**Case presentation:**

We present two cases of classic ameloblastoma in children. We describe in detail the patients’ characteristics, treatment processes, and follow-up result. The bone formation and reconstruction in the lesion area after fenestration decompression and curettage are recorded at every clinic review. The surgical details and principles of curettage and decompression are also described and discussed. The two patients have good bone shape recovery and no recurrence.

**Conclusions:**

Children are in the growth and development period and possess an extremely strong ability of bone formation and reconstruction. Based on the principles of minimally invasive and functional preservation, we believe that curettage combined with decompression can be the first choice for treating AM in children, especially for mandibular lesions.

## Introduction

According to the World Health Organization (WHO) [1] classification of oral and maxillofacial tumors, orofacial neoplasms are classified into benign and malignant odontogenic tumors, benign and malignant maxillofacial bone and cartilaginous tumors, benign and malignant soft tissue lesions, Fibro-osseous and Haemato-lymphoid tumors. Ameloblastoma (AM) is the most common benign odontogenic tumor, accounting for approximately 10% of all oral lesions. AM mainly occurs in the jaw near the molars in young adults, which is more often detected in the mandible than maxilla, especially the mandibular body and mandibular angle. There is no significant gender-dependent difference [2]. The growth of the tumor is slow, and there are no apparent symptoms at the initial stage. However, after gradual development, AM can cause jaw bulges and deformity. Moreover, AM can lead to occlusal disorder, lower lip numbness, and pathological fracture, affecting normal mastication, breathing, and swallowing function. AM can be classified into several subtypes: such as classic, unicystic, peripheral/extraosseous, and metastatic AM, according to the new classification of WHO in 2017 [3]. The surgical management of orofacial tumors is presented with considerable challenges because of their location near the sensitive areas of the face. The surgical manipulating modalities are decided by maxillofacial surgeons according to the clinical examination and collated diagnostic information, taking into consideration fast healing, minimal scarring, and least deformity [4]. For classic AM (also called conventional AM), the mainstream treatment is partial jaw resection or even segmental resection, and free vascularized iliac bone flap or fibula repair is performed in the area of the bone defect [5]. This method has the advantages of a strong radical cure and accurate effect, which can avoid recurrence as much as possible. However, the postoperative functional and psychological influence can not be ignored. Especially for children in the period of growth and development, an osteotomy is often challenging to be accepted by their parents [6–8]. Ameloblastoma in this group of patients presents a special challenge in the management because of the need to offer a conservative treatment that would take into consideration the continuing development and growth of the affected jaws [9]. With the rise of functional surgery, an increasing number of surgeons tend to treat ameloblastoma by conservative means to retain the original jaw architecture and function, despite the higher recurrence rate after treatment [5, 10]. Our previous retrospective study showed that the effective rate of fenestration decompression combined with secondary curettage(FDSC) in the treatment of multicystic ameloblastoma was 71.19%, and the effective rate in the treatment of unicystic ameloblastoma was 93.02% [11]. Therefore, we tend to use similar conservative treatment options for pediatric patients.

Up to now, only very few studies have reported the selection and evaluation of treatment methods for juvenile AM [8, 12, 13]. Particularly, no report has been carried out on patients aged 10 and younger. In the present work, we reported two children with classic AM, who were treated by curettage combined with decompression. Two patients were admitted for the first time, and their age was less than or equal to 10 years old when receiving surgery. After follow-up, the effect of this regimen remained good, which was reported as follows.

## Case presentation

### Case 1

A 10-year-old male came to see the doctor because of right facial swelling with local discomfort for 5 months. An enhanced CT examination of the maxillofacial region at the local hospital suggested the possibility of AM in the right mandible.

**Fig. 1:**
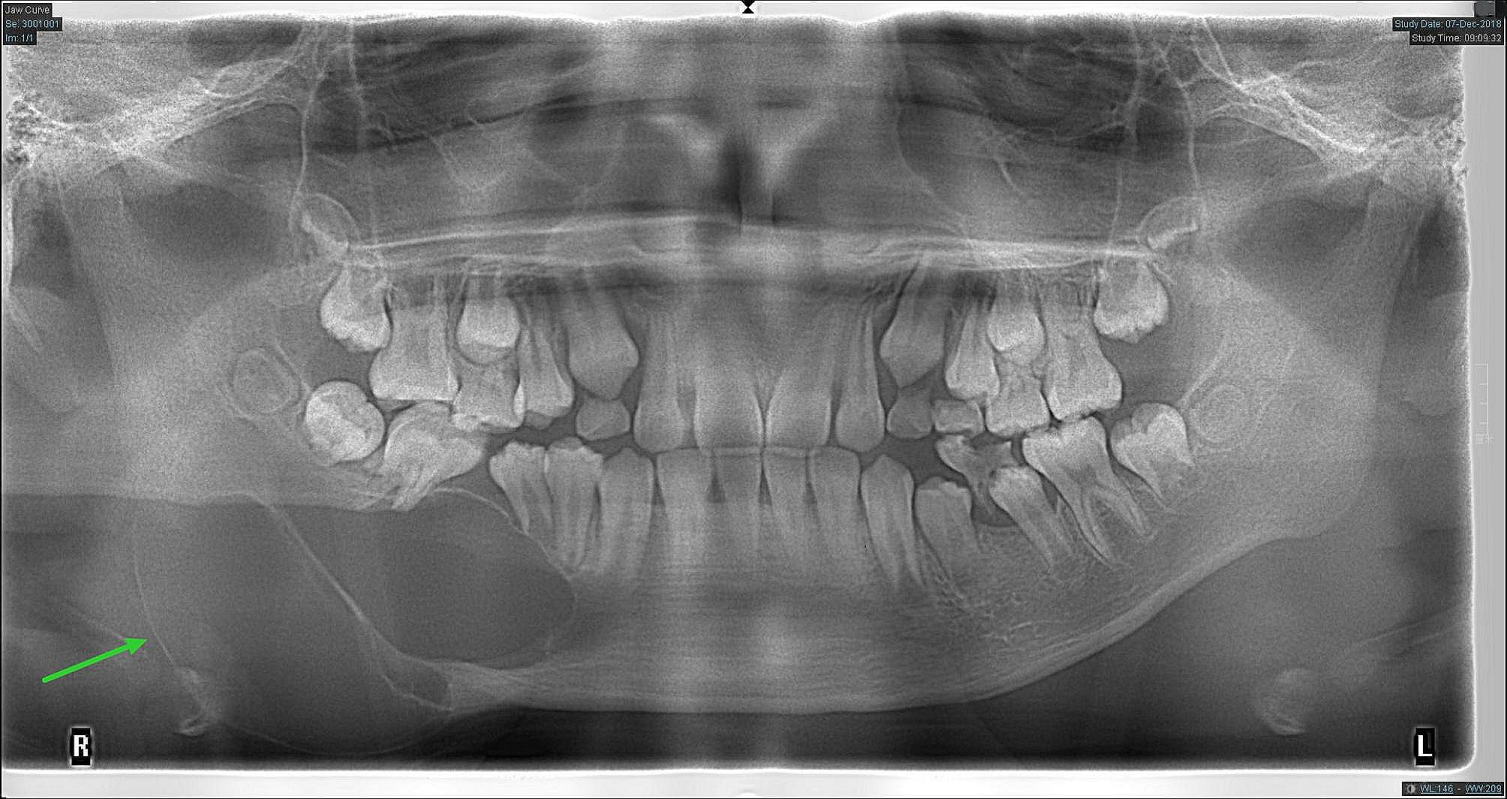
Preoperative panoramic radiography showing a typical AM in the right mandible

The right face and mandibular angle area were dilated, the skin color and temperature were normal, the mouth opening was limited, and there was no numbness in the lower lip. The right mandibular posterior tooth vestibular sulcus and ascending branch were seen in the mouth, the palpation was hard, there was no ping-pong-like sensation, the mucous membrane was intact, and the tooth 46 was II °loose. As shown in Fig. 1, the preoperative panoramic radiography revealed a typical AM: multilocular cystic low-density shadow of different sizes, overlapping each other, clear boundaries, and AM-involving teeth with truncated root resorption.

**Fig. 2:**
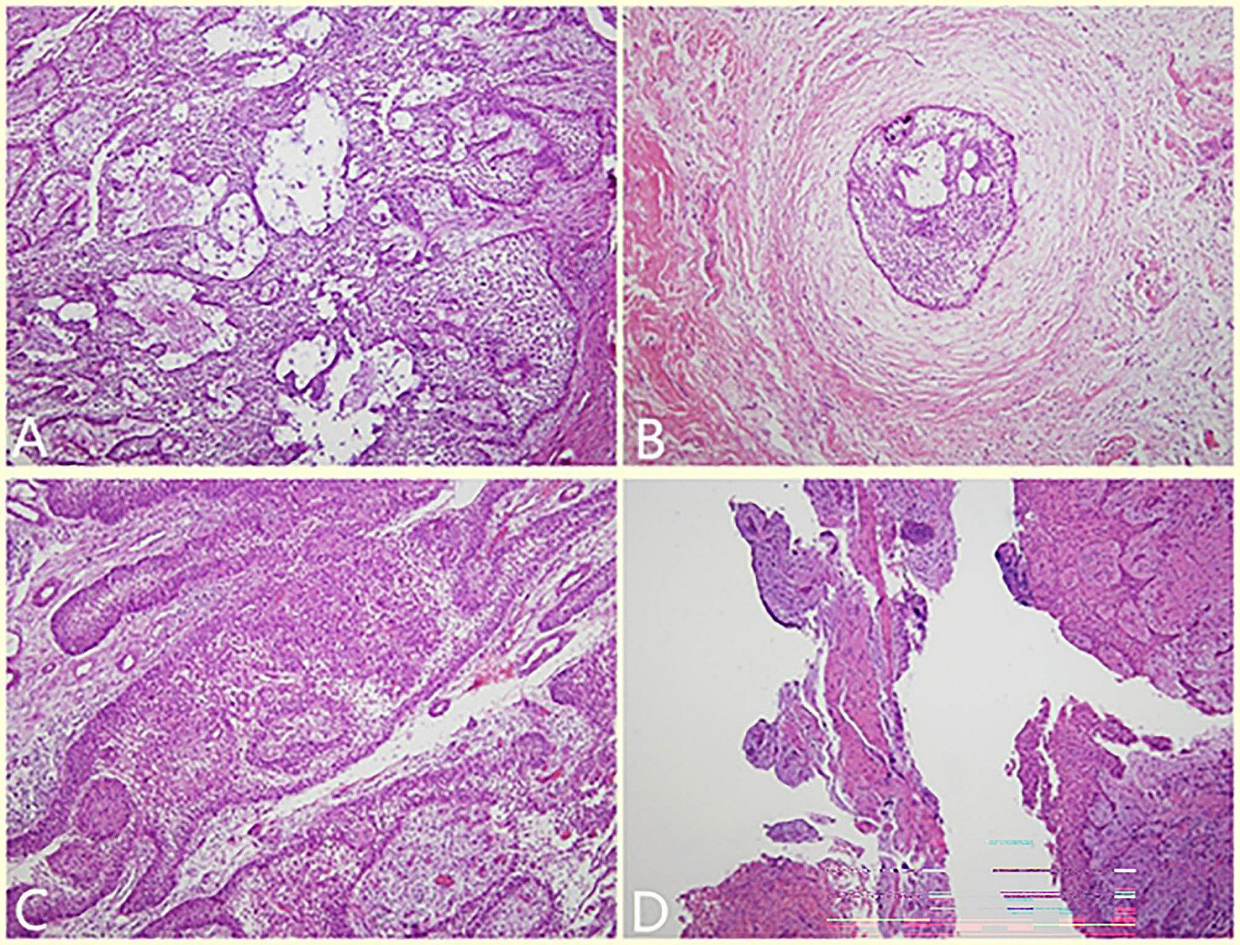
Histopathological photos (HE staining,100X). **A**, Tumor cells arranged in a follicular or plexiform pattern with abundant cells, evidenced by the active proliferation of interstitial fibrous tissue. **B**, Pathology after treatment showed obvious degeneration, with only partially odontogenic epithelium seen. **C**, Tumor cells arranged in a follicular or plexiform pattern, revealing the appearance of classic AM. **D**, Pathology after treatment showed hyperplastic fibers and mucosal epithelium, and a small amount of reticular hyperplasia of the squamous epithelium was observed focally

The patient had no contraindication of surgery, and on December 20, 2018, decompression of the right mandibular tumor was performed under general anesthesia. During the operation, teeth 45 and 46 were extracted, and the mucoperiosteum was excised on the surface of the bulging buccal area. Part of the bone wall was removed, and the tumor was opened to form a window. The tumor was solid, and a small amount of tissue near the opening window was excised and sent for frozen pathological analysis, indicating mandibular AM with abundant cells. The iodoform gauze was packed in the window without a suture. The postoperative paraffin-embedded section (Fig. 2A) indicated a classic AM with abundant cells, evidenced by the active proliferation of interstitial fibrous tissue, basal-like cells in the outer layer, star-reticular cells in the inner layer, and tumor cells arranged in a follicular or plexiform pattern.

About 1 week after the operation, the iodoform gauze was removed from the patient in the outpatient clinic, and a plug device was made and installed in the prosthetic department. After that, the reexamination was conducted regularly in the outpatient clinic. During the reexamination, we mainly focused on whether there was any abnormality in the soft and hard tissues of the operation area, adjustment was made on the plug device, and panoramic radiography was carried out to evaluate the shrinkage of the cyst.

**Fig. 3:**
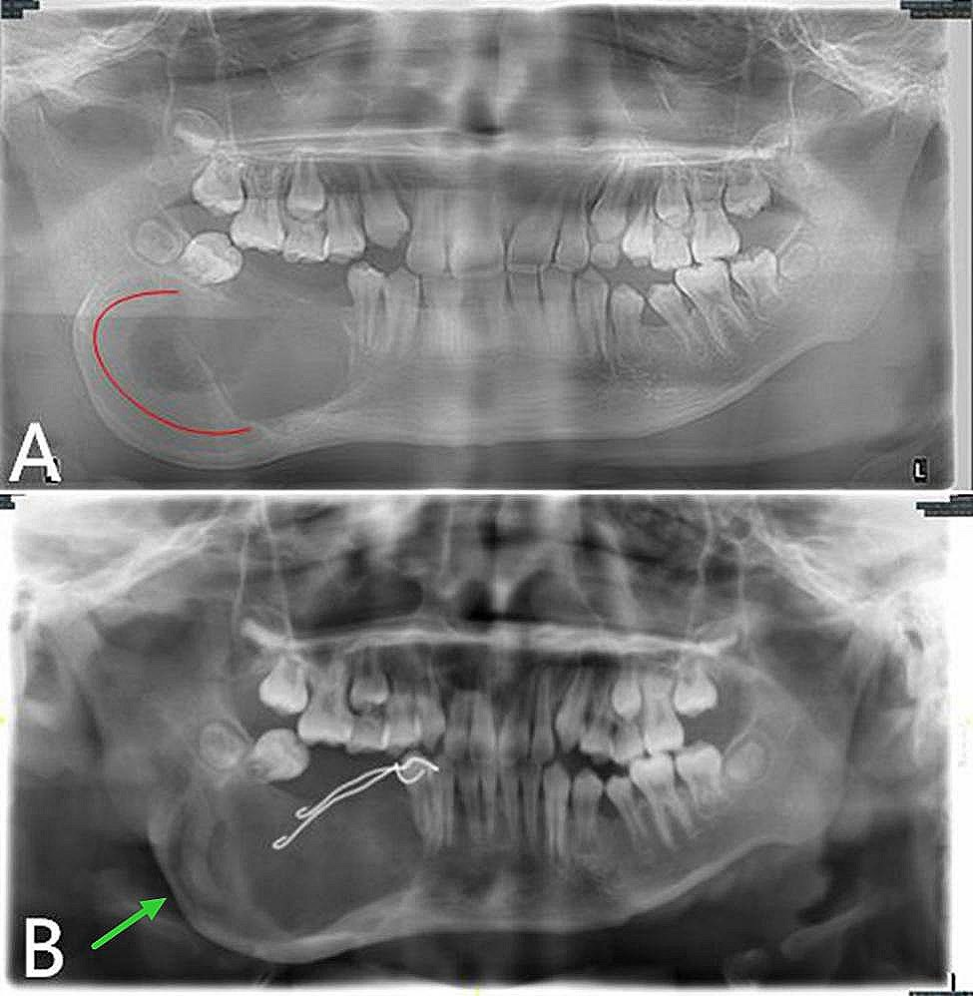
Panoramic oral radiogram. **A**, 3 months after the operation. **B**, 6 months after the operation. The original osteogenic area (**A**, red curve) disappear compared with **B**

The reexamination 6 months after the operation showed that the low-density shadow in the jaw became more significant compared with that 3 months ago, and the original osteogenic area partially disappeared (Fig. 3). Local recurrence was considered. Then on June 27, 2019, the right mandibular tumor was cured by intraosseous curettage combined with decompression under general anesthesia. During the operation, the original opening window was enlarged, the tumor was completely eliminated along the bone wall, and the ball drill was used to grind off part of the bone wall. Special attention was paid to protect the inferior alveolar neurovascular bundle. The intraoperative frozen pathological analysis confirmed AM. To stop bleeding, the bone cavity was packed with iodoform gauze. The postoperative paraffin-embedded section showed the same result as the one before operation.

**Fig. 4:**
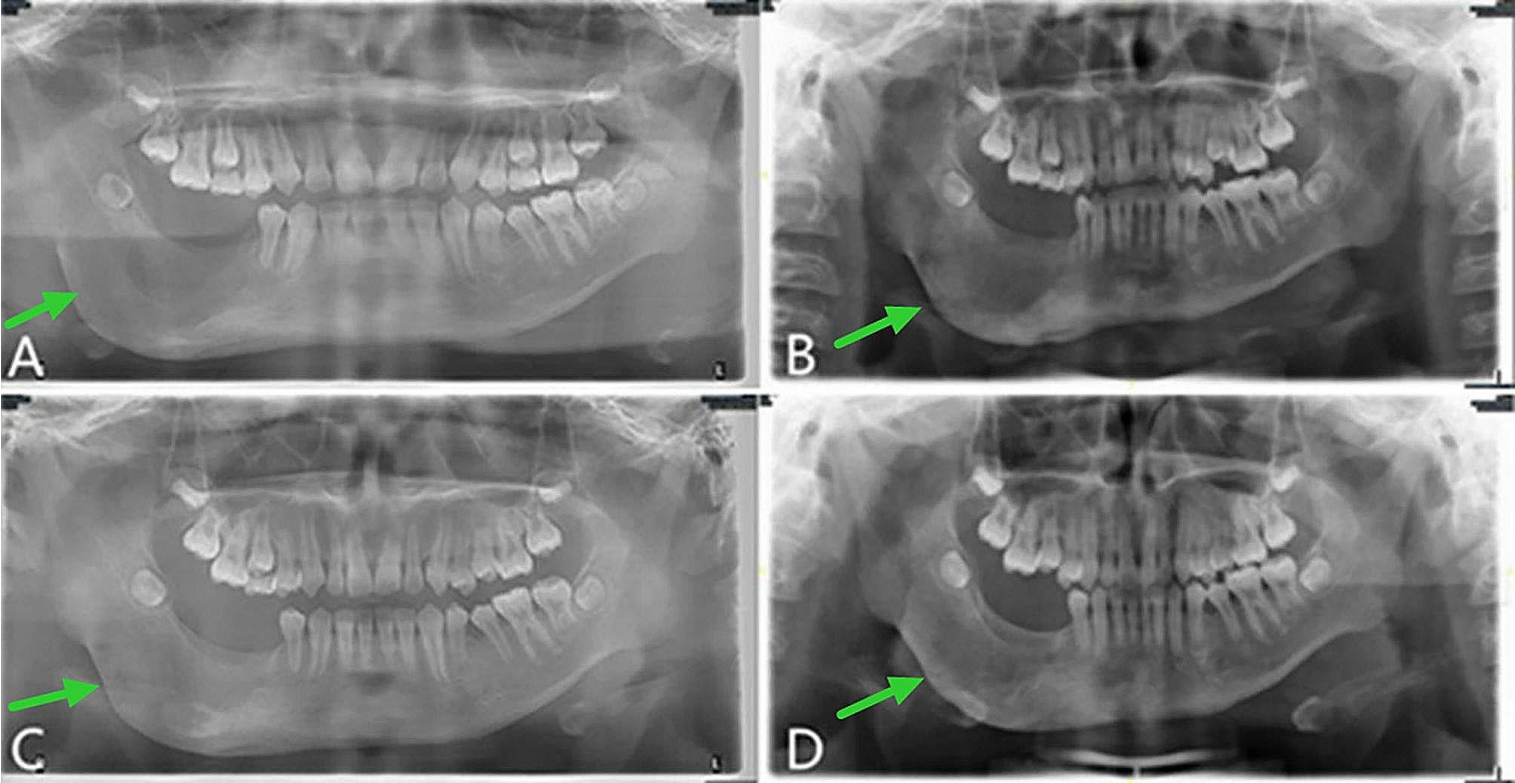
Follow-up panoramic oral radiogram after the second operation, displaying the continuous bone remodeling. **A**, 3 months postoperative. **B**, 4 months postoperative. **C**, 6 months postoperative. **D**, 10 months postoperative

**Fig. 5:**
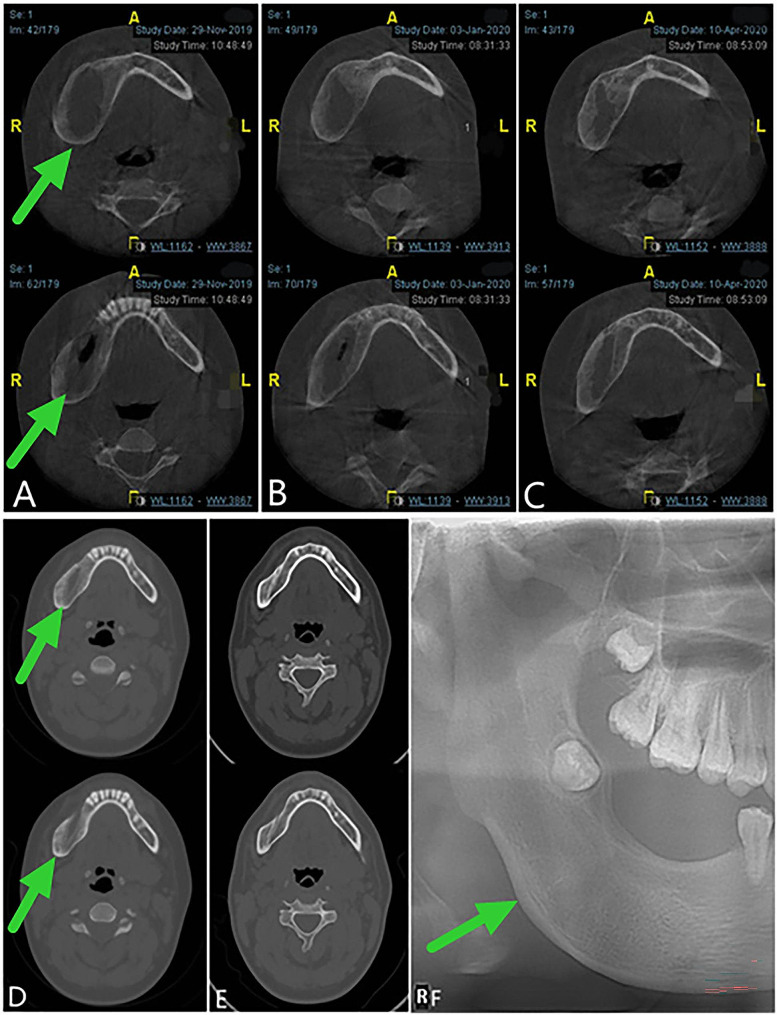
Follow-up images. **A**, **B** and **C**, Cone beam computed tomography showing 5, 7,10 months respectively after the second operation. **D** and **E**, Computed tomography view displaying the continuous osteogenic remodeling 4 and 15 months after the window is closed. **F**, Panoramic oral radiogram demonstrating 15 months after the surgery to close the window

After the operation, the patient wore the plug device, and the outpatient examination was carried out regularly (Fig. 4). At 10 months after the second operation (April, 2020), the patient was readmitted to the hospital for surgery to close the window. During the procedure, if the fenestration remained and the bottom was shallow, an incision was designed along the perimeter of the fenestration. The mucoperiosteum of the fenestration was excised, the bone surface was exposed, and the soft tissue in the bone cavity was removed. Combined with the medical history, the local lesions were consistent with AM, with fibrous tissue tumor-like hyperplasia and hyaline degeneration. The mucoperiosteum was released on both sides of the window, and the adjacent flap was turned to close the window. The postoperative paraffin-embedded section showed a fibrous capsule wall-like tissue, lined with stratified squamous epithelium, which had odontogenic features (Fig. 2B). Combined with the medical history, the local lesions were consistent with AM. The patient was followed up for 15 months after the window was closed. No recurrence was found. Osteogenic remodeling could be seen in the bone cavity. The shape of the original bulging jaw returned to normal (Fig. 5).

### Case 2

A 9-year-old female patient was diagnosed with a painless mass in the right mandible for 1 year. The patient was required to undergo a tonsillectomy 1 year ago, and a right mandibular mass was accidentally discovered during the CT scan. There was no conscious pain, numbness, and other symptoms. Before the patient came to our hospital, she had an opened window for decompression and wore a plug in the local hospital, which was ineffective.

**Fig. 6:**
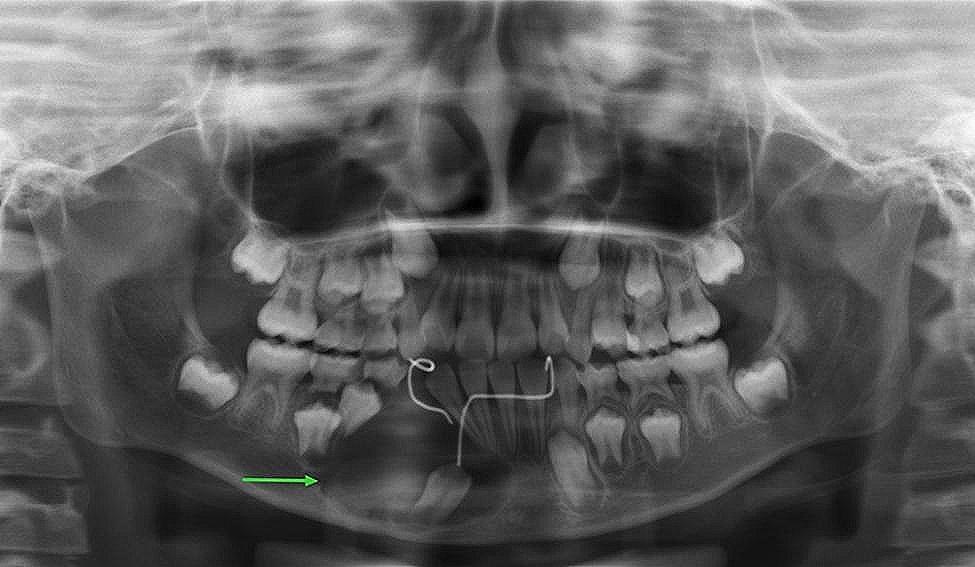
Preoperative panoramic radiography

The right mandibular body was slightly distended, the mouth opening was normal, and there was no numbness in the lower lip. Mixed dentition was found in the mouth, vestibular sulcus was distended from 42 distal to 46 mesial in the right mandible, and a palpable mass of about 2 × 3 cm was observed. The preoperative panoramic radiography was shown in Fig. 6, indicating that the tumor was cystic, with a notch-like bony alba line on the edge. The boundary was clear, the lower part was adjacent to the lower edge of the mandible, the tooth germ of tooth 43 was seen inside, and the teeth on both sides of the tumor were squeezed and displaced. The preoperative diagnosis was AM of the right mandible.

After excluding contraindications, the patient underwent curettage and decompression of the right mandibular mass under general anesthesia on March 14, 2019. During the operation, the deciduous teeth 4 and 5 loosened were extracted, and a window with a long diameter of 2.5 cm was expanded in the vestibular sulcus along with the most apparent buccal tumor bulge. First, the cystic wall at the opening window was removed and sent for rapid pathological analysis, and the report confirmed the “right mandible” AM. Then, the remaining cystic wall was further scraped, the low-impacted tooth 43 was extracted, and the tumor was removed entirely. The bone cavity was filled with iodoform gauze to stop bleeding, and the window remained open without a suture. The postoperative paraffin-embedded section (Fig. 2C) illustrated basal-like cells in the outer layer, star-reticular cells in the inner layer, and tumor cells arranged in follicular plexiform, revealing the appearance of classic AM.

**Fig. 7:**
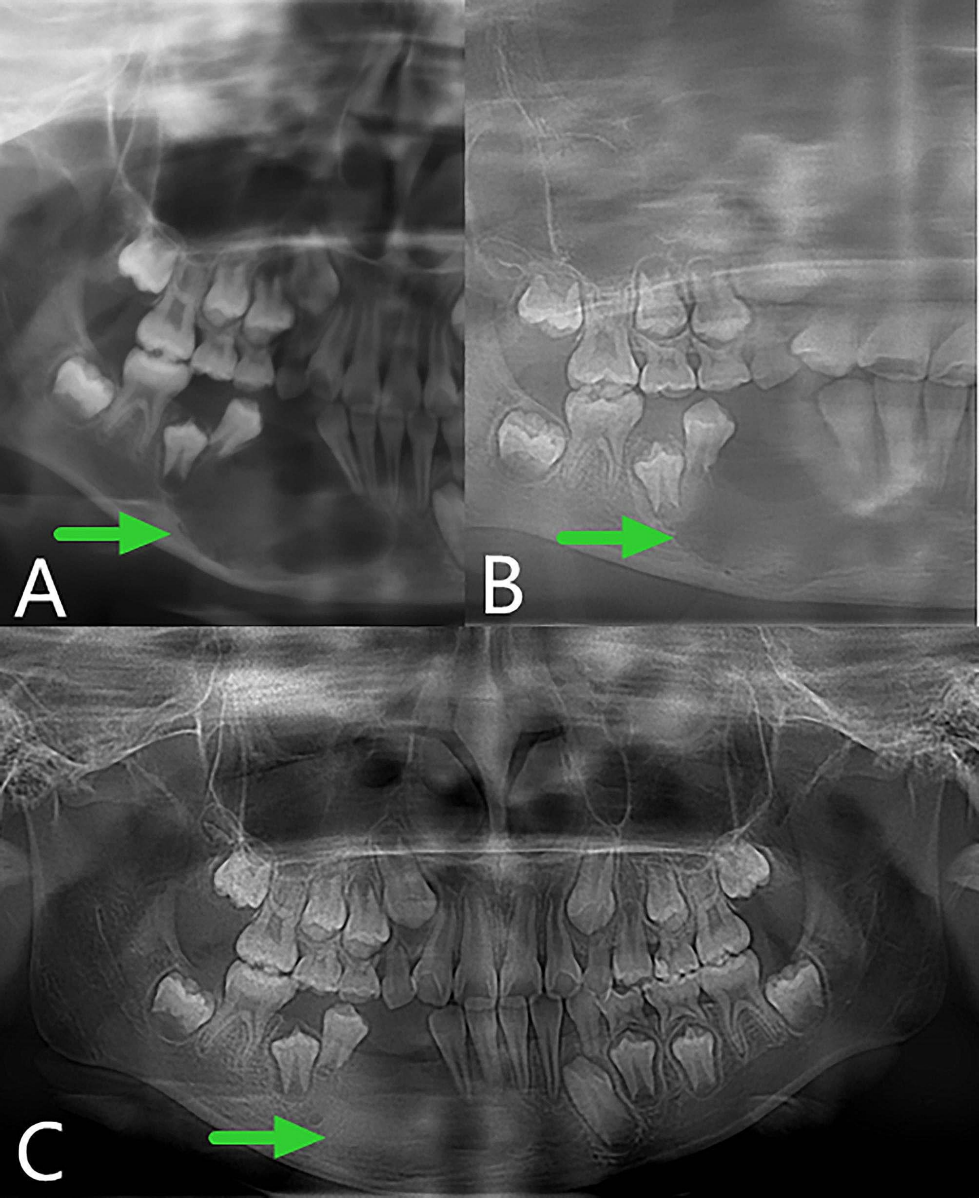
Postoperative panoramic oral radiogram. **A**, 1 week. **B**, 2 months. **C**, 4 months

About 1 week after the operation, the iodoform gauze was removed from the patient in the outpatient clinic, and a plug device was made and installed in the prosthetic department. After that, the reexamination was carried out at the outpatient clinic regularly. During the reexamination, the main focus was on whether there was any abnormality in the soft and hard tissues of the operation area. The plug device was adjusted, and panoramic oral radiographs were taken to evaluate the shrinkage of the cyst. Figure 7 showed that the cyst cavity was significantly reduced during the follow-up visit, and a considerable amount of bone formed at 4 months after the operation.

**Fig. 8:**
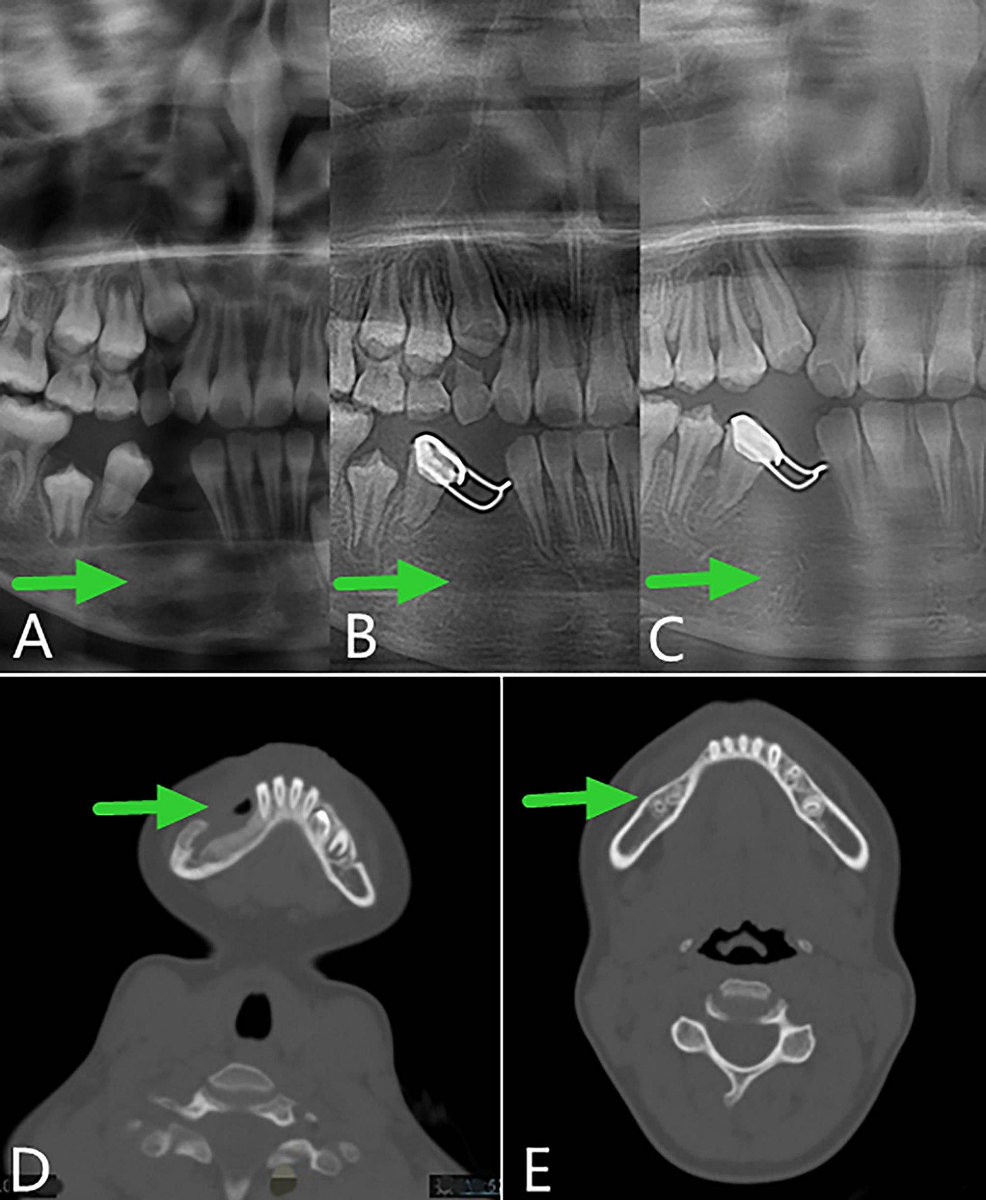
Panoramic radiography and computed tomography. **A**, **B** and **C**, Panoramic oral radiogram showing 2 months, 1 year and 2 years respectively after secondary curettage and fenestration closure. **D** and **E**, Computed tomography view displaying 4 months and 28 months after the curettage and fenestration

After evaluation by our team, the child underwent stage II surgery in our hospital 4 months after the first operation (2019-08-01) to close the window, and the lesion was further clarified. During the operation, we found that the bone cavity in the opening window became shallow and narrow. The incision was made along the edge of the opening window, and the mucosa of the bony cavity and the surface part of the oral mucosa were excised together. The mucoperiosteal flaps on both sides were released, and the opening window was closed and sutured. The postoperative paraffin-embedded section indicated hyperplastic fibers and mucosal epithelium, and a small amount of reticular hyperplasia of the squamous epithelium was observed focally (Fig. 2D). Combined with the medical history, AM was not excluded. The child was followed up for 28 months after surgery (28 months after curettage and fenestration and 24 months after secondary curettage and fenestration closure). The operation area continued to have good osteogenic remodeling, and the bone appearance was improved without recurrence (Fig. 8).

## Discussion

Ameloblastoma is known to be uncommon in children and young adults and studies show that its prevalence varies due to factors ranging from race and location where the study was done, the age limit chosen for the pediatric population and duration of study [14]. The global incidence of ameloblastoma is 0.5 cases/million people, with 10–15% of cases occurring in the pediatric population, reaching up to 25% in Africa and Asia [15]. Nwoga’s retrospective observational study [16] shows children in the study (1–10 years) constituted 13.5% (5) while adolescents (11–18 years) made up 86.5% (32) of all the 37 ameloblastoma observed in children and adolescents. This study (13.5%), combines with those of Arotiba et al. [17] (2.5%), Okoh et al. [18] (9.3%), Iyogun et al. [19] (5.6%), and Ajayi et al. [20] (8.5%), provide us with data on the proportion of patients with ameloblastoma less than 10 years old. It seems that patients with ameloblastoma under the age of 10 are not as rare as imagined, reflecting regional and ethnic differences. Koraitim et al. [13] find that ameloblastoma tends to occur in the older age group (mean = 12.6years). Their findings are consistent with other studies, which show that about 90% of ameloblastoma occurs in children older than 11 years [21, 22]. By reviewing the post-1970 literature, Ord et al. [8] have concluded that the mean age of AM children is 14.3 years (Western) and 14.7 years (Africa), and less than 10% of those patients are 10 years and younger.

For the treatment of AM, the pathological subtype is one of the essential references. Ameloblastoma classification has been narrowed to conventional(classic) ameloblastoma, unicystic and extraosseous/peripheral types. The solid/multicystic type was eliminated because most conventional ameloblastomas show cystic degeneration with no biological differences. The desmoplastic type was left under the histopathological subtype (follicular, plexiform, acanthomatous, granular cell, basaloid and desmoplastic) rather than as a separate entity. The current consensus is that one-stage decompression + two-stage curettage or curettage combined with grinding of the bone wall can achieve better therapeutic effects for the unicystic type [23]. The recurrence rate of extraosseous/peripheral type is also very low after the tumor resection is extended to 0.5 cm outside the tumor [24]. However, there are many controversies and room for improvement in the treatment of classic AM. Marginal jaw resection, partial jaw resection, and even segmental resection with simultaneous free ilium or vascularized ilium or fibula repair have been reported [6, 25]. For adult classic AM, radical resection and reconstruction are effective approaches and can avoid recurrence to the greatest extent possible. However, for pediatric patients with the same lesions, it is more challenging to make treatment decisions because we need to consider the age, growth, and development of children and adolescents, donor site complications, etc. Radical surgeries often destroy the oral and maxillofacial function and aesthetics, and have a certain impact on patients’ growth and development, physical and mental health. Although the recurrence rate is higher than that of radical surgery, another point of view regards that recurrence is not the most important consideration in case of children as even recurrent cases were shown to require less aggressive treatment than that would have been performed for initial lesion [26].

Due to the low incidence, the management of AM patients aged under 10 is inconclusive. Ord et al. [8] have advocated the use of osteotomy to treat children with classic or recurrent AM after the first treatment, which is also the primary method for adult AM. Their experience holds that unless the lesions are found in the early stage and the size of the tumor is small enough to ensure that the continuity of the mandible can be maintained after marginal resection of the 1-cm bone boundary outside the tumor, the marginal resection that preserves the lower edge of the mandible still has the potential for recurrence. Of the 11 pediatric patients they have summarized, six are treated with curettage, three have a recurrence, and one patient is lost to follow-up. Moreover, three patients receive marginal excision, one patient has a recurrence, and one patient is lost to follow-up. In addition, two patients who receive partial jaw resection and bone graft reconstruction have no recurrence. All patients with recurrence undergo partial jaw resection, one patient undergoes fibula reconstruction, and no recurrence is found during follow-up. It is worth noting that the age of these reported pediatric patients ranges from 12 to 19, and there are no patients aged under 10. Rong Yong et al. [27] review a total of 104 cases of primary pediatric ameloblastomas treated in their hospital. The surgical methods used in their cohort suggest that pediatric ameloblastomas are often managed by decompression or direct curettage (84.62%) vs. bone resection (15.38%). They conclude that the maximum tumor diameter, root resorption, and bone cortex/soft tissue invasion are risk factors for recurrence of pediatric ameloblastomas, while there is no significant association between recurrence and surgical method of treatment (*P* = .74). Peng X et al. [28] propose sequential method (Stage I decompression + Stage II endoscopic-assisted curettage + Stage III osteotomy) for treatment of juvenile large cystic classic AM. The results show that stage I fenestration decompression + stage II endoscopically assisted curettage is effective for most patients. A few patients with recurrence need to be combined with multiple endoscopically assisted curettage. A very small number of patients with recurrence are recommended to undergo stage III osteotomy and repair.

Given the high recurrence rate of curettage and the impact of osteotomy on growth, development, and psychology of children, we aim to use curettage combined with decompression in recent years to treat patients with classic AM who meet the indications, and satisfactory results have been achieved. Previous research from our team has demonstrated that fenestration decompression combined with secondary curettage (FDSC) may serve as a routine, safe, effective and appropriate surgical treatment plan for AM patients with large lesions [11]. The superiority of this approach was particularly evident in the treatment responses of the two patients aged under 10.

Classic AM is easy to relapse when only curettage is used in the treatment because AM has a certain degree of bone infiltration and multilocularity. Unlike the cyst wall with complete boundaries, it is difficult to eradicate all tumor cells by curettage. Residual tumor cells are easy to recur. However, the following two goals will be achieved if decompression is performed simultaneously. ① Changing the environment in which tumor cells survive. The osteoclast-promoting cytokines secreted by the epithelial cells of the cyst wall are released into the oral environment. The changes in the microenvironment of the cyst cavity cause changes in the tumor epithelium. Different pathological changes, such as keratinization and hyalinization, appear, and the tumor epithelium gradually degenerate, eventually, transforms into the oral mucosa epithelium [29]. This point was also confirmed by the pathological changes of the two patients in this study (show in Fig. 2). ② Reducing the pressure of tumor invasion into bone tissue and rebalancing osteoclast and bone remodeling. Under the dynamic effect of mandibular growth and reconstruction, the rate of osteogenesis is much greater compared with tumor osteoclasts (especially in children). When a new balance is achieved (the jaw bone lesions are no longer reduced), two-stage surgical curettage can be used to obliterate the tumor.

More attention should be paid to several points as follows when using this approach. (1) We should ensure that the opening window is large enough that the contents of the cyst cavity can be fully drained. (2) Unlike unicystic AM and keratinizing cyst, decompression is not adequate for classical AM. During the operation, on the premise of ensuring that the vital nerve and blood vessels are not damaged, it is necessary to scrape as much as possible the visible tumor components. Fenestration decompression was only used in the first operation of the two patients described in this study. Case 1 recurred, and case 2 had no effect. (3) For multilocular lesions, the bony septum should be opened entirely, by which the multilocular structure becomes a single-chamber cystic cavity. Otherwise, large lesions may remain during curettage. In addition, postoperative drainage is not smooth, reducing the jaw cavity will be limited, and postoperative recurrence will occur. In the present study, case 1 recurred after the first operation. Besides the reason that only fenestration was performed, there was another critical reason. The reexamination radiograph revealed that the bone septum was not sufficiently removed, resulting in poor drainage, and residual tumor cells in the dead space could not communicate directly with the oral environment. (4) Protecting the integrity of the periosteum. The periosteum acts as a physiological and anatomical barrier, and violence should be avoided during the curettage process. If the periosteum ruptures, the tumor may enter the soft tissue, resulting in the recurrence of implanted soft tissue.

## Conclusions

Children are in the growth and development period and possess an extremely strong ability of bone formation and reconstruction. We believe that decompression combined with curettage could be the first choice for treating AM in children, especially for mandibular lesions. In the future, it is necessary to accumulate more cases for research and follow-up throughout the life cycle.

## Data Availability

The datasets used and/or analysed during the current study are available from the corresponding author on reasonable request.

## List of abbreviations

AM: Ameloblastoma

## References

[1] Wright JM, Vered M (2017). Update from the 4th Edition of the World Health Organization Classification of Head and Neck tumours: odontogenic and maxillofacial bone tumors. Head Neck Pathol.

[2] Palanisamy JC, Jenzer AC. Ameloblastoma. Treasure Island (FL). StatPearls Publishing; 2023. 31424749

[3] Soluk-Tekkeşin M, Wright JM. The World Health Organization Classification of Odontogenic Lesions: A Summary of the Changes of the 2017 (4th) Edition. Turk Patoloji Derg. 2018;34(1). 10.5146/tjpath.2017.01410 28984343

[4] Mohammed K, Aldelaimi A, Enezei H, Aldelaimi T (2021). Tumors of Craniofacial Region in Iraq (Clinicopathological Study). J Res Med Dent Sci.

[5] Neagu D, Escuder-de la Torre O, Vázquez-Mahía I, Carral-Roura N, Rubín-Roger G, Penedo-Vázquez Á, Luaces-Rey R, López-Cedrún JL (2019). Surgical management of ameloblastoma. Review of literature. J Clin Exp Dent.

[6] Luo HY, Li TJ (2009). Odontogenic tumors: a study of 1309 cases in a Chinese population. Oral Oncol.

[7] Kreppel M, Zöller J (2018). Ameloblastoma-Clinical, radiological, and therapeutic findings. Oral Dis.

[8] Ord RA, Blanchaert RH, Nikitakis NG, Sauk JJ (2002). Ameloblastoma in children. J Oral Maxillofac Surg.

[9] Almajid EA, Alfadhel AK (2019). Management of large pediatric ameloblastoma: conservative approach with 4-years follow up. Oral Maxillofac Surg Cases.

[10] Hendra FN, Natsir Kalla DS, Van Cann EM, de Vet HCW, Helder MN, Forouzanfar T (2019). Radical vs conservative treatment of intraosseous ameloblastoma: systematic review and meta-analysis. Oral Dis.

[11] Wu K, Luo H, Yuan Z, Wang Y, Qin X, He J (2022). Clinical evaluation of fenestration decompression combined with secondary curettage for ameloblastoma of the jaw: retrospective radiographic analysis. BMC Oral Health.

[12] Okechi UC, Akpeh JO, Chukwuneke FN, Saheeb BD, Okwuosa CU, Obi DI, Ogbozor BE (2020). Ameloblastoma of the jaws in children: an evaluation of cases seen in a tertiary hospital in South-Eastern Nigeria. Ghana Med J.

[13] Koraitim M, Medra AM, Salloum AM, Shehata EA (2022). Pediatric Aggressive Benign Mandibular tumors: clinical features and management. J Craniofac Surg.

[14] Anyanechi CE, Saheeb BD (2014). A review of 156 odontogenic tumours in Calabar, Nigeria. Ghana Med J.

[15] Effiom OA, Ogundana OM, Akinshipo AO, Akintoye SO (2018). Ameloblastoma: current etiopathological concepts and management. Oral Dis.

[16] Nwoga MC (2021). Ameloblastoma in Children and adolescents: a seven-year study in Enugu, Nigeria. J Paediatr Dent Res Pract.

[17] Arotiba GT, Ladeinde AL, Arotiba JT, Ajike SO, Ugboko VI, Ajayi OF (2005). Ameloblastoma in Nigerian children and adolescents: a review of 79 cases. J Oral Maxillofac Surg.

[18] Okoh DS, Akinshipo AO, Butali A, Omitola OG, Sigbeku OF, Soyele OO, Osunde OD, Taiwo AO, Ibikunle AA, Omeje KU (2020). Descriptive Epidemiology of Odontogenic Tumors in Nigeria: an African oral Pathology Research Consortium Multicenter Study. Niger J Clin Pract.

[19] Iyogun CA, Omitola OG, Ukegheson GE (2016). Odontogenic tumors in Port Harcourt: South-South geopolitical zone of Nigeria. J Oral Maxillofac Pathol.

[20] Ajayi OF, Ladeinde AL, Adeyemo WL, Ogunlewe MO (2004). Odontogenic tumors in Nigerian children and adolescents- a retrospective study of 92 cases. World J Surg Oncol.

[21] Zhang J, Gu Z, Jiang L, Zhao J, Tian M, Zhou J, Duan Y (2010). Ameloblastoma in children and adolescents. Br J Oral Maxillofac Surg.

[22] Chawla R, Ramalingam K, Sarkar A, Muddiah S (2013). Ninety-one cases of ameloblastoma in an Indian population: a comprehensive review. J Nat Sci Biol Med.

[23] Oginni FO, Stoelinga PJ, Ajike SA, Obuekwe ON, Olokun BA, Adebola RA, Adeyemo WL, Fasola O, Adesina OA, Akinbami BO (2015). A prospective epidemiological study on odontogenic tumours in a black African population, with emphasis on the relative frequency of ameloblastoma. Int J Oral Maxillofac Surg.

[24] Milman T, Ying GS, Pan W, LiVolsi V (2016). Ameloblastoma: 25 year experience at a single Institution. Head Neck Pathol.

[25] Hendra FN, Van Cann EM, Helder MN, Ruslin M, de Visscher JG, Forouzanfar T, de Vet HCW (2020). Global incidence and profile of ameloblastoma: a systematic review and meta-analysis. Oral Dis.

[26] Huang IY, Lai ST, Chen CH, Chen CM, Wu CW, Shen YH (2007). Surgical management of ameloblastoma in children. Oral Surg Oral Med Oral Pathol Oral Radiol Endod.

[27] Yang R, Tang Y, Zhang X, Liu Z, Gokavarapu S, Lin C, Ren Z, Zhou Y, Cao W, Ji T (2019). Recurrence factors in pediatric ameloblastoma: clinical features and a new classification system. Head Neck.

[28] Peng X, Zhang C, Han R, Wang D, Liu C, Du R, Gao T, Zhang K (2023). Clinical study of sequential treatment of large cystic ameloblastoma in minors. J Pract Stomatol.

[29] Yang Z, Liang Q, Yang L, Zheng GS, Zhang SE, Lao XM, Liang YJ, Liao GQ (2018). Marsupialization of mandibular cystic ameloblastoma: retrospective study of 7 years. Head Neck.

